# Comprehensive Evaluation of Electronic Medical Record System Use and User Satisfaction at Five Low-Resource Setting Hospitals in Ethiopia

**DOI:** 10.2196/medinform.4106

**Published:** 2015-05-25

**Authors:** Binyam Tilahun, Fleur Fritz

**Affiliations:** ^1^Institute of Medical InformaticsUniversity of MünsterMünsterGermany; ^2^Department of Health InformaticsUniversity of GondarGondarEthiopia

**Keywords:** electronic medical record, evaluation, low-resource settings, Ethiopia, DeLone and MacLean model

## Abstract

**Background:**

Electronic medical record (EMR) systems are increasingly being implemented in hospitals of developing countries to improve patient care and clinical service. However, only limited evaluation studies are available concerning the level of adoption and determinant factors of success in those settings.

**Objective:**

The objective of this study was to assess the usage pattern, user satisfaction level, and determinants of health professional’s satisfaction towards a comprehensive EMR system implemented in Ethiopia where parallel documentation using the EMR and the paper-based medical records is in practice.

**Methods:**

A quantitative, cross-sectional study design was used to assess the usage pattern, user satisfaction level, and determinant factors of an EMR system implemented in Ethiopia based on the DeLone and McLean model of information system success. Descriptive statistical methods were applied to analyze the data and a binary logistic regression model was used to identify determinant factors.

**Results:**

Health professionals (N=422) from five hospitals were approached and 406 responded to the survey (96.2% response rate). Out of the respondents, 76.1% (309/406) started to use the system immediately after implementation and user training, but only 31.7% (98/309) of the professionals reported using the EMR during the study (after 3 years of implementation). Of the 12 core EMR functions, 3 were never used by most respondents, and they were also unaware of 4 of the core EMR functions. It was found that 61.4% (190/309) of the health professionals reported over all dissatisfaction with the EMR (median=4, interquartile range (IQR)=1) on a 5-level Likert scale. Physicians were more dissatisfied (median=5, IQR=1) when compared to nurses (median=4, IQR=1) and the health management information system (HMIS) staff (median=2, IQR=1). Of all the participants, 64.4% (199/309) believed that the EMR had no positive impact on the quality of care. The participants indicated an agreement with the system and information quality (median=2, IQR=0.5) but strongly disagreed with the service quality (median=5, IQR=1). The logistic regression showed a strong correlation between system use and dissatisfaction (OR 7.99, 95% CI 5.62-9.10) and service quality and satisfaction (OR 8.23, 95% CI 3.23-17.01).

**Conclusions:**

Health professionals’ use of the EMR is low and they are generally dissatisfied with the service of the implemented system. The results of this study show that this dissatisfaction is caused mainly and strongly by the poor service quality, the current practice of double documentation (EMR and paper-based), and partial departmental use of the system in the hospitals. Thus, future interventions to improve the current use or future deployment projects should focus on improving the service quality such as power infrastructure, user support, trainings, and more computers in the wards. After service quality improvement, other departments (especially inter-dependent departments) should be motivated and supported to use the EMR to avoid the dependency deadlock.

## Introduction

### Background

Electronic medical record (EMR) systems are increasingly being implemented in hospitals to achieve the following six aims of improved care: (1) safety, (2) effectiveness, (3) patient centeredness, (4) timeliness, (5) efficiency, and (6) quality [1]. However, many EMR systems which are technically sound for developers and healthcare managers, face resistance from users and may end up in failure [2].

Measuring success of an information system is difficult because success does not have a common explicit definition [3], and is dependent on expectations. The agreed hypothesis to say an information system is successful is when the implemented system is accepted to be used by the end user and the users are satisfied with the system. As a result, a number of researchers suggested that user satisfaction and system use are the primary determinants of user adoption, and therefore are suitable to measure information system success [4-7]. Mazzoleni et al describe health professionals' satisfaction towards EMR system as “essential to the survival” of the system [8]. The different implementation projects which were reported as failed have often been those in which the end users were dissatisfied or the core system functions were not properly used [9].

Even though most health professionals generally perceive that technology can help eliminate the burden of paper-based documentation and the unavailability of patient data in critical situations, they also get easily dissatisfied when an introduced system or support does not meet their expectations [10]. Pare et al [11] assessed the factors in clinical information system implementation success and reported on the necessity of identifying risk factors and involving health professionals starting from the development and pilot phase to avoid failure. Many factors affect the adoption of EMR systems and they vary within the system users, hospital setting, and the type of system in use [12,13].

EMR systems are also increasingly being incorporated into the healthcare organizations of developing countries that do not have well-developed infrastructure and well-trained technical personnel to use and manage the systems. As outlined by Sood [14], the determinant factors that affected the information system success in those settings might be different from factors in developed countries. Hence, rigorous evaluation studies on different health information system implementation projects in those settings are necessary to understand the critical success and failure factors. To date, since only a few reports are available [15], this study was conducted to fill this gap by evaluating the use and user satisfaction of an EMR system implemented in Ethiopia.

### Rationale

In the current health sector development plan of Ethiopia, strengthening the health management information system and incorporating a computerized health management information system (HMIS) are priority policy plans to ensure health service quality and equity [16]. As a result, an HMIS documentation package was implemented to standardize the patient documentation and reporting systems in all health facilities of the country [17]. The implementation of this standardized documentation system was mandatory and a prerequisite to implement the EMR system to make sure that the paper workflow is in line with the EMR.

In 2009, the Ministry of Health, with support of the Tulane University Technical Assistance Project in Ethiopia (TUTAPE), started the development and implementation of a comprehensive EMR system for hospitals called SmartCare. The system was deployed in 5 hospitals in Addis Ababa [18] and other hospitals in regional cities. In 2013, the Ministry of Health adapted the system as a national EMR for all hospitals, and planned to scale it up to further hospitals and regions [19].

Even though the system developers claimed it the best EMR, it had not been thoroughly evaluated by a neutral investigator. As explained by Joaquin [20] and Fraser [21], information system projects, especially those which were developed by NGO’s, need thorough independent evaluation prior to scale-up to determine if system expansion is both worthwhile and feasible. Hence, this study conducted by an independent and neutral investigator, filled this gap by identifying the main factors which needed to be addressed before a costly expansion.

### Objectives

The main objectives of this study are (1) to assess the current EMR use rate among health professionals, (2) to assess the use level of core EMR functions, (3) to determine the user satisfaction level of health professionals, and (4) to identify determinants factors of user satisfaction towards the EMR system in the study hospitals. The study was conducted in accordance with the Guidelines for Good Evaluation Practices in Health Informatics (GEP-HI) [22] and reported based on the Statement on Reporting of Evaluation Studies in Health Informatics (STARE-HI) [23].

### Study Context

#### Organizational Setting

This evaluation study was conducted in 5 hospitals in Ethiopia. All are government hospitals located within a 15 km radius in Addis Ababa, the capital city of Ethiopia. Of the 5 hospitals, one is an 80-bed children and mothers care specialized hospital with inpatient and outpatient clinics, 2 are 300-bed teaching referral hospitals with different specialized clinics, and the remaining 2 are 200-bed general hospitals with both inpatient and outpatient services. All of the hospitals implemented the HMIS in 2009, and started EMR implementation in 2011 [24].

#### System Detail and System in Use

SmartCare is a portable, integrated EMR system that is currently used by three African countries (Zambia, Ethiopia, and South Africa), and presumably is the largest EMR system in use in Africa [25]. The system was designed in Africa to be robust in environments with limited infrastructure. The system also offers a touch screen interface to minimize the learning curve.

**Figure 1:**
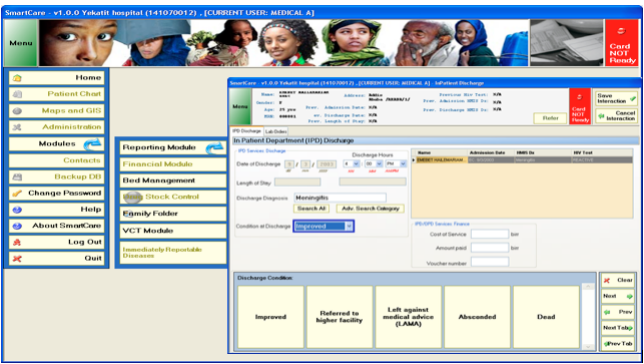
Screenshot of the SmartCare EMR system currently implemented in Ethiopian hospitals. The main modules are listed on the left side of the image. The main modules have sub-modules that will be displayed upon clicking. The screenshot shown is displayed when "bed management" is clicked.

This comprehensive EMR system has different components (modules) that can be used in the various units of healthcare facilities (Figure 1). The main modules of SmartCare include registration, outpatient department, inpatient (to admit, follow, and discharge patients in wards), tuberculosis, pediatrics, HIV/AIDS (to manage patients in antiretroviral therapy clinics), antenatal care, postpartum, pharmacy, drug stock control, laboratory (to store and send laboratory results to the requesting clinic), eHMIS (to generate monthly, quarterly, and annual reports), and finance. Currently all but the financial module are implemented and used in the hospitals of this study.

Installation of the network, server infrastructure, and the EMR system at all hospital sites was conducted by TUTAPE. After implementation, 5 day-long onsite user training sessions were provided to all health professionals of each hospital. Additionally, TUTAPE computer and network experts are responsible to provide continuous on-call service for technical assistance during system failure.

On average, the SmartCare system has been in use in the 5 hospitals of this study since 2011. In parallel, the paper-based medical record system is also still in use which means that the health professionals are expected to document both on paper and within the EMR system. The plan of the government is to expand the system to the other 127 existing hospitals in the country after the pilot testing. Additionally, the government is training health informatics professionals to support the health management information system and implementation of EMR in the country [26].

## Methods

### Study Design

A quantitative, cross-sectional study design based on a validated questionnaire was used to assess the use pattern, user satisfaction level, and determinant factors of SmartCare in 5 government hospitals located in Addis Ababa. To better understand the use and challenges of the system, we also assessed the current method of documentation on the EMR server and the fluctuation levels of power access in the study hospitals. The selected hospitals were chosen because the EMR system had been implemented for 3 years. Additionally, as 2 are teaching hospitals and 3 are general hospitals, representative hospital types were included.

### Theoretical Background

This study was conducted based on the DeLone and MacLean (D&M) information system success evaluation model [4], a validated and the most commonly used information system success evaluation method among the informatics community [4]. The basic dimensions in this model are system quality, information quality, service quality, system use, user satisfaction, and net benefit.

For this evaluation, we chose 5 factors from the D&M model that are relevant for user satisfaction and use rate evaluation, by excluding net benefit. Instead of net benefit, user background was included as a determinant factor to be tested because many researchers reported it as a determinant factor especially in low literacy working environments [27]. Additionally, we assessed the level of use of core EMR functions since they have been found to be a main factor of user satisfaction [28,29].

### Participants and Sample Size

The participants of this study, health professionals across the 5 study hospitals, were categorized into the following four groups (1) physicians (doctors and health officers), (2) nurses (clinical and midwifery), (3) lab and pharmacists (laboratory and pharmacy professionals), and (4) HMIS (health data entry and management secretaries, information system officers, and data clerks). The sample size of 422 participants was calculated assuming a 95% Cl and 10% non-response rate. All health professionals, who were selected by a simple random sampling technique among their professional category and who also served for >6 months in the hospitals, were approached to complete the questionnaire.

### Study Flow

This study began in January, 2014 after obtaining ethical clearance. The first step was to choose data collectors from each hospital and familiarize them with the objective and methodology of the research. Seven data collectors were chosen and trained on how to collect the questionnaire and the level of support they should give to avoid bias. The questionnaires were distributed to the participants by visiting them in their offices, mostly during the afternoon. To motivate participants, we provided one Samsung Galaxy III phone as a reward, by a lottery method, to all of the participants who fully completed the questionnaire. Data collection took place over a one-month period.

### Outcome Measure and Evaluation Criteria

The outcome measures and corresponding evaluation criteria are shown in Textbox 1.

**Outcome measures and evaluation criteria.**

Measure

- EMR use rate
  - Measured by the proportion of respondents who are currently using the system and server log analysis of current patient data documentation in the EMR system.
- Use rate of core EMR functions
  - Measured by the frequency of use of 12 core functionalities of the implemented EMR system.
- User satisfaction level
  - Evaluated by a median of 5 different user satisfaction measurement items based on a 5-point Likert scale (1-strongly agree to 5-strongly disagree).

- Factors determining user satisfaction
  - Measured by a binary logistic regression analysis of all user characteristic and organization factors.

### Data Acquisition and Measurement

A questionnaire was developed based on standardized and previously validated instruments. The questions were divided into three categories. The first category, on the user background, had 15 questions about general socio-demographic data, computer training, and current use of the EMR system. Some of them were adapted from Mahmood et al [12], Lawrence and Low [30], and Igbara and Nachman [31]. The second category was designed to measure the perceived system quality, information quality, service quality, satisfaction, and expectation towards future benefits. To assess system quality, 7 items were used, whereas 10 were used to assess information quality, 9 to assess service quality, 5 to assess user satisfaction questions, and 3 to assess expectations towards future benefit. The items were adapted from Seddon et al [32] and Doll et al [33]. For the service quality, we added additional setting-specific questions to reflect the power interruptions and the computer access challenges faced in the study hospitals. The third category contained 12 questions on core EMR functions which were adapted from Moustafa et al [9] with amendments from EMR officers on the main core functionalities of the system.

A pretest of the questionnaire was conducted in a hospital that was not part of the study in which 5 physicians, 8 nurses, 3 lab/pharmacists, and 5 HMIS staffs participated. Based on the pretest results, 2 questions were amended for wording as they were reported to be unclear from a health professional’s perspective. The reliability of the items was evaluated with Cronbach’s alpha, and the values were all above .84, indicating satisfactory reliability of the questionnaire.

### Data Analysis

Descriptive statistics were performed to describe the characteristics of the participants, EMR use rate, and user satisfaction. Binary logistic regression analysis was used to identify determinant factors of user satisfaction among the study participants.

The selected dependent variable for this study was “user satisfaction”. In the questionnaire, participants were asked to rate their satisfactions on a 5-point Likert scale. Median and interquartile ranges (IQRs) with percentages were used. In the cross tabulation of our data, we found that responses of “very satisfied” and “very dissatisfied” were very low. Consequently, for the logistic regression, the 5-item scales were merged into two groups from “very satisfied and satisfied” and "very dissatisfied and dissatisfied" to “satisfied” “dissatisfied”, respectively. After this dichotomization, the determinant factors were analyzed using binary logistic regression. All analyses were performed using SPSS Software version 22.

### Ethical Statement

Ethical approval was granted by the Institutional Review Board (IRB) of the University of Gondar and the Addis Ababa City Administration Health Bureau. Permission for data collection was also obtained from each of the hospitals. The participants were informed about the study, its importance, and confidentiality of the information collected, as well their right to leave the study at any time. Written consent was obtained from participants in a form provided with the questionnaire and the procedure was approved by the IRB.

## Results

### Socio-Demographic Characteristics

Out of the 422 participants of this study, 96.2% (406/422) completed the questionnaire. Of all the questionnaires, 7 were not completed and 9 were not returned. The mean age of the participants was 34 years (SD 8.5). Of all the participants, 53.4% (217/406) were males, and the majority of the participants were nurses (43.3%, 176/406), followed by physicians (20.4%, 83/406), HMIS staff (18.2%, 74/406), and laboratory and pharmacy staff (18%, 73/406). The participants had a mean work experience of 8.6 years (SD 7.2) in the current hospital. The detailed socio-demographic characteristics of the respondents are shown in Table 1.

**Table 1:** Frequencies of the socio-demographic characteristics of the study participants (n=406).

Characteristics		Frequency	Relative frequency, %
**Age of respondents, years**			
	<30	161	39.7
	31-40	136	33.5
	41-50	84	20.7
	>50	25	6.2
**Sex**			
	Male	217	53.4
	Female	189	46.6
**Work experience in current hospital, years**			
	<5	166	41.1
	5-15	172	42.6
	<15	66	16.3
**Professional category**			
	Physicians	83	20.4
	Nurses	176	43.3
	Lab and pharmacists	73	18.0
	HMIS staff	74	18.2
**Part-time job**			
	Yes	106	26.1
	No	300	73.9

### Study Findings

#### EMR Use Pattern and Related Characteristics

Among the respondents, 76.1% (309/406) started using the system immediately after implementation and user training. In this context, 'use' refers to a complete use of the EMR to document patient information, in addition to the patient card. Among them, the major proportion of users were HMIS staff 20.7% (64/309), followed by nurses 44.0% (136/309), laboratory and pharmacy staff, and physicians 18.4% (57/309). However, during the data collection, only 31.7% (98/309) of the professionals reported to use the system with the majority being HMIS staff (54.0%, 53/98), followed by nurses (33.6%, 33/98), lab and pharmacy staff (33.6%, 33/98), and physicians (7.1%, 7/98). Those who completely stopped using the EMR reported that they were using the computers for other purposes such as browsing the Internet, word processing, while others returned them to the store.

#### Training, Information Technology Qualification, and EMR Experience

In terms of training, 64.0% (260/406) participated in the EMR user training and 60.6% (246/406) had been previously trained on the HMIS implementation. Almost half of the staff (47.2%, 192/406) responded to having a “reasonable information technology (IT) qualification”. The majority (76.1%, 309/406) did not have previous EMR experience, 20.4% (83/406) reported to having individual computer access in the office, and of those, the majority were HMIS staff (55.4%, 46/83). The others shared the computer access with 2 people (11.5%, 47/406), 3 people (16.7%, 68/406), 4 people (17.7%, 72/406) and the majority shared with more than 5 people (57.8%, 235/406), mainly nurses (94.8%, 223/235) (Table 2).

**Table 2:** Training, information technology (IT) qualification, experience, and current EMR use status of physicians, nurses, laboratory, pharmacy, and HMIS staff in the study participants (n=406).

Characteristics		n (%)NursesLaboratory& PharmacyHMIS			
		Physicians	Nurses	Laboratory & Pharmacy	HMIS
**Computer access in hospital**					
	Individual	11 (20.4)	12 (9.0)	14 (26.9)	46 (71.9%)
	For 2 practitioners	4 (7.4)	25 (18.7)	11 (21.2)	7 (10.9)
	For 3 practitioners	13 (24.4)	33 (24.6)	14 (26.9)	8 (12.5)
	For 4 practitioners	23 (42.6)	42 (31.3)	5 (9.6)	2 (3.1)
	For >5 practitioners	3 (5.6)	223 (16.4)	8 (15.4)	1 (1.6)
**HMIS training**					
	Yes	22 (26.5)	131 (74.4)	35 (47.9)	58 (78.4)
	No	61 (73.5)	45 (25.6)	38 (52.1)	16 (21.6)
**IT qualification**					
	No qualification	18 (21.7)	43 (24.4)	37 (50.7)	6 (8.1)
	Reasonable qualification	58 (69.9)	82 (46.6)	25 (34.2)	27 (36.5)
	Good qualification	7 (8.4)	51 (29.0)	11 (15.1)	41 (55.4)
**SmartCare training**					
	Yes	28 (33.7)	120 (68.2)	45 (61.6)	67 (90.5)
	No	55 (66.3)	55 (31.3)	28 (38.4)	7 (9.5)
**Another EMR experience**					
	Yes	35 (42.7)	16 (9.1)	25 (34.2)	20 (27.0)
	No	47 (57.3)	160 (90.9)	48 (65.8)	54 (73.0)
**SmartCare use since implementation**					
	Yes	57 (68.7)	136 (77.3)	52 (71.2)	64 (86.5)
	No	26 (31.3)	40 (22.7)	21 (28.8)	10 (13.5)
**Current SmartCare use**					
	Yes	7 (12.1)	33 (24.3)	5 (9.4)	53 (81.5)
	No	51 (87.9)	103 (75.7)	48 (90.6)	12 (18.5)

#### Usage Pattern From Server Log Analysis

The observations of the patient chart in the registration department of the hospitals showed that on average 184,594 patients per hospital have paper-based records. Out of those, 58.7% (108,450/184,594) were also available in the EMR system database. However, only 4.8% (5244/108,450) of those patients had a documented main diagnosis patient history in the EMR server.

In terms of infrastructure, hospitals on average had 61 computers and one server for the EMR system. Four of the hospitals had one or more IT staff members, however, they were not specifically hired for the EMR system. Rather, they primarily worked for the statistics office and they took the EMR system work as their secondary task. The number of IT staff, computers, and the number of medical records in the hospital paper and server databases is shown in Table 3.

**Table 3:** Information technology (IT) professionals, reported number of EMR-dedicated computers, and patient records the Addis Ababa study hospitals from January-February, 2014. Most of the information available in the paper-based record system was not registered on the computer. Patient registration in the card room was done by data clerks while the main diagnosis was written by physicians or nursing assistants.

Characteristics	Hospital 1	Hospital 2	Hospital 3	Hospital 4	Hospital 5
Number of IT staffs	1	2	0	1	1
Number of computers for the EMR system	73	61	55	66	51
Number of patients with paper-based records	222,937	179,327	71,985	171,292	277,421
Number of patients registered in the EMR system	199,866	155,967	55,644	95377	35,398
Number of patients who have a main diagnosis in the EMR	7841	4848	4500	5323	3721

#### Main Reported Reasons of Not Using the System

**Figure 2:**
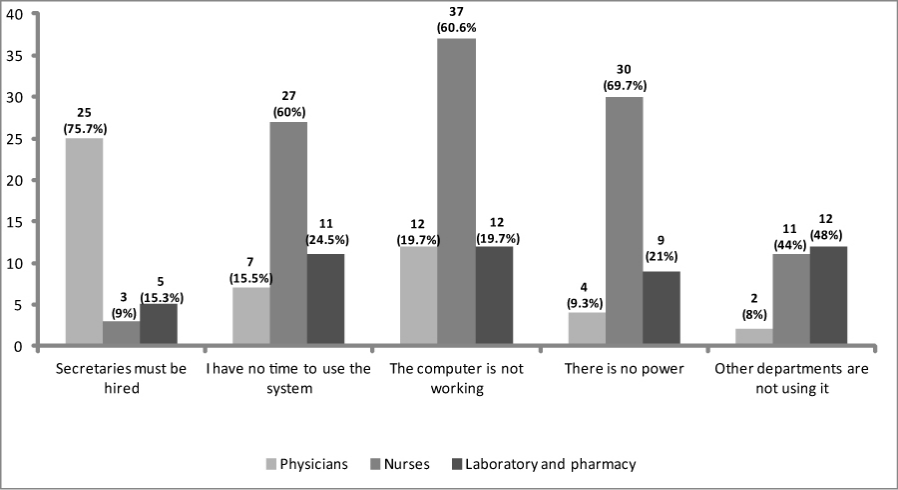
Main reported reasons for not using the EMR system by physicians, nurses, and laboratory and pharmacy staff (n=197).

An open-ended item question was provided to assess the main reasons for not using the system (Figure 2). Among the respondents who reported that they did not have the time to use it, the majority were physicians (75.7%, 25/33). Among the respondents who reported that the main reason for not using the system was that the computers were not working were nurses (60.6%, 37/61), while 69.7% (30/43) reported that power fluctuations in their department were the main obstacle. Of the respondents who reported that “the other departments were not using the system” was the main reason for not using the EMR, 48% (12/25) were laboratory and pharmacy staff.

#### Use of the Main EMR Components in the Study Hospitals

Participants were also assessed on the use of the main components of the EMR as shown in Table 4. The most frequently used functionalities of the respondents were “find patients with certain characteristics” (45.6%, 141/309), and “produce patient summary” (45.9%, 142/309). The detailed use of the EMR components by the various categories of health professionals is shown in Table 4.

**Table 4:** Use of the basic EMR components (n=309).

Component	Physicians, n (%) N=56			Nurses, n (%) N=136			Lab and pharmacists, n (%) N=51			HMIS staff, n (%) N=63		
	U^a^	R^b^	F^c^	U	R	F	U	R	F	U	R	F
Find patient with certain characteristics	5 (8.9 )	7 (12.5)	44 (78.5)	19 (13.9)	31 (22.7)	86 (63.2)	9 (17.6)	40 (78.4)	2 (3.9)	19 (30.1)	35 (55.5)	9 (14.2)
Create notes (history and physical exam)	45 (80.3)	6 (10.7)	5 (8.9)	51 (37.5)	65 (47.7)	20 (14.7)	10 (19.6)	4 (7.8)	37 (72.5)	23 (36.5)	33 (52.3)	7 (11.1)
Enter order (lab, radiology)	42 (75.0)	8 (14.2)	6 (10.7)	59 (43.3)	61 (44.8)	16 (11.7)	9 (17.6)	40 (78.4)	2 (3.9)			
Review/obtain lab and radiology results	4 (7.1)	9 (16.0)	43 (76.7)	27 (19.8)	64 (47.0)	45 (33.0)						
Update diagnosis	42 (75.0)	7 (12.5)	7 (12.5)	23 (16.9)	90 (66.1)	23 (16.9)						
Review currently received medications	6 (10.7)	5 (8.9)	45 (80.3)	20 (14.7)	88 (64.7)	28 (20.5)	7 (13.7)	6 (11.7)	38 (74.5)			
Write prescriptions	43 (76.7)	6 (10.7)	7 (12.5)	53 (38.9)	58 (42.6)	25 (18.3)						
Admit a patient	6 (10.7)	6 (10.7)	44 (78.6)	61 (44.8)	47 (34.5)	28 (20.5)						
Refer a patient	9 (16.0)	46 (82.1)	1 (1.9)	24 (17.6)	86 (63.2)	26 (19.11)						
View/schedule appointment for a patient	5 (8.9)	9 (16.0)	42 (75.0)	75 (55.1)	30 (22.0)	31 (22.7)				55 (87.3)	6 (9.5)	2 (3.1)
Communication using SmartCare's communication	10 (17.8)	5 (8.9)	41 (73.2)	59 (43.3)	33 (24.2)	44 (32.3)	8 (15.6)	6 (11.7)	37 (72.5)	58 (92.0)	2 (3.1)	3 (4.7)
Produce patient summary reports	10 (17.8)	5 (8.9)	41 (73.2)	62 (45.5)	25 (18.3)	48 (35.3)				3 (4.7)	7 (11.1)	53 (84.1)

^a^Unaware of the function (U)

^b^Rarely used the function (R)

^c^Frequently used the function (F)

#### EMR Satisfaction and Expectation for Future Benefit

Among the participants, 64.4% (199/309) responded to be dissatisfied with the use of the implemented EMR system. Of those dissatisfied, 24.6% (49/199) were physicians and 52,7% (105/199) were nurses. The participants responded with a strong disagreement towards the statements “The system helps me finish my task faster” (median=5, IQR=1) and “The system has a positive effect on quality of care” (median=5, IQR=1). Of all the professionals, 67.9% (210/309) preferred the paper-based record to the EMR system. Overall, the median satisfaction level was at the range of “Disagree” (median =4, IQR=1). The overall median responses with IQRs and percentages are shown in Table 5.

**Table 5:** Median satisfaction level of the study participants (n=309).

Characteristics of EMR Satisfaction	Physicians		Nurses		Laboratory and pharmacy		HMIS	
	n (% DA^a^) n=57	mean (IQR^b^)	n (% DA) n=136	mean (IQR)	n (% DA) n=52	mean (IQR)	n (% DA) n=64	mean (IQR)
SmartCare help me to finish my work faster	49 (85.9)	5 (1)	89 (65.4)	4 (2)	46 (88.4)	4 (0)	2 (3.1)	2 (1)
EMR Improves my productivity	47 (82.4)	4 (0)	52 (38.2)	3 (1)	41 (78.8)	4 (0)	1 (1.5)	3 (1)
I prefer the EMR than the paper record	13 (22.8)	4 (1)	50 (36.7)	3 (2)	7 (13.4)	2 (1)	0 (0.0)	2 (2)
System has positive impact on quality of care	48 (84.2)	5 (1)	107 (78.6)	4 (0)	42 (80.7)	5 (1)	2(3.1)	2 (1)
Overall, I am satisfied with the EMR system	49 (85.9)	5 (1)	105 (77.2)	4 (0)	44 (84.6)	5 (0)	2 (3.1)	2 (1)
Category median score (95% CI), median (IQR)	5 (1)		4 (1)		4 (2)		2 (1)	
Over all median score (95% CI), median (IQR)	4.0 (1.0)							

^a^Disagree (DA)

^b^Interquartile range (IQR)

#### Perceived System, Information, and Service Quality

**Figure 3:**
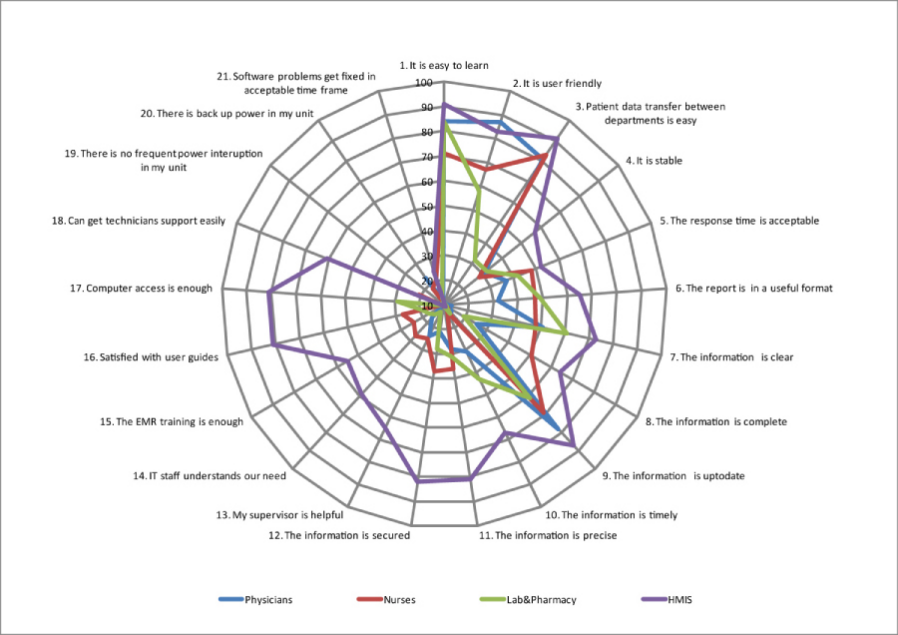
Perceived system, information, and service quality of the study (n=309). The numbering on the label is to show in which category the criteria belong (1-5=System quality; 6-12=Information quality; 13-21=System quality). The main axis is the reported percentage. As shown in the figure, HMIS staff give more positive responses than phycisians and nurses .

The respondents indicated an agreement with the statement that the implemented EMR system had an acceptable quality with an overall median score in the range of “agree” (median=2, IQR=0.5). Of the health professionals, 77.6% (240/309) found the system easy to learn, 61.1% (189/309) user friendly, 58.2% (180/309) stable, and 55.9% (173/309) found the response time acceptable. Overall, HMIS staff perceived the system to have more quality when compared to the other professional categories (median=2, IQR=1). All the criteria to measure system quality (1-5), information quality (6-12), and service quality (13-21) with percentages are shown in Figure 3.

The participants of this study also agreed with the statement that the information quality of the implemented system was acceptable with an overall median range of “agree” (median=2, IQR=1.0) with more acceptance within HMIS staff (median=1, IQR=1.0) and less agreement by physicians (median=4, IQR=2.0). Of all the participants, 93.5% (289/309) found the output of the system useful, and 76.6% (237/309) also found the information on the modules sufficient for their clinical practice. Only 42.3% (131/309) reported that they felt secure when using the system.

More dissatisfaction was reported with the service quality with an overall median score in the range of “disagree” (median=4.5, IQR=1.5). Of the respondents, 56.6% (175/309) believed their immediate supervisors were not helpful in using the EMR system, 71.8% (222/309) thought the IT support staff did not understand their needs, and 61.8% (191/309) believed the training given was not adequate. Additionally, 66.9% (207/309) responded that they could not get a computer in the ward during patient treatment, 66.0% (204/309) were unhappy with the computer technicians support, 73.4% (227/309) were also unhappy with the frequent power interruptions, and among them, 58.2% (180/309) responded that their department was not backed up by the standby generator.

#### Expectation Towards Future Benefit

Expectations of the respondents about the benefit of the EMR system for the patient, professionals, and the hospital were also assessed (Table 6). The majority of the respondents who never use the EMR system (91.7%, 89/97) and the current users (53.6%, 52/97) expect that the EMR system will be beneficial. Of the respondents who used to use the system, 45.3% (140/309) reported that the EMR system will be beneficial to the hospital. An independent sample *t* test revealed a statistically significant difference between “those who never use the system” and “those who used to use the system” (*P*<.001), but not significant between “those who used to use the system” and “current users”.

**Table:** Expectations of future benefits of EMR users and non-users (n=406).

Characteristic	Those who never use EMR, n (%A^a^), n=97	Those who used to use, n (%A), n=309	Current users, n (%A), n=98
I expect EMR to benefit patients in the future	89 (91.7)	140 (45.3)	52 (53.0)
I expect EMR to benefit staff in the future	80 (82.4)	130 (42.0)	46 (46.9)
I expect EMR to benefit the hospital in the future	94 (96.9)	120 (38.9)	50 (51.0)

^a^Agree

#### Power Interruption Rate in the Study Hospitals

**Figure 4:**
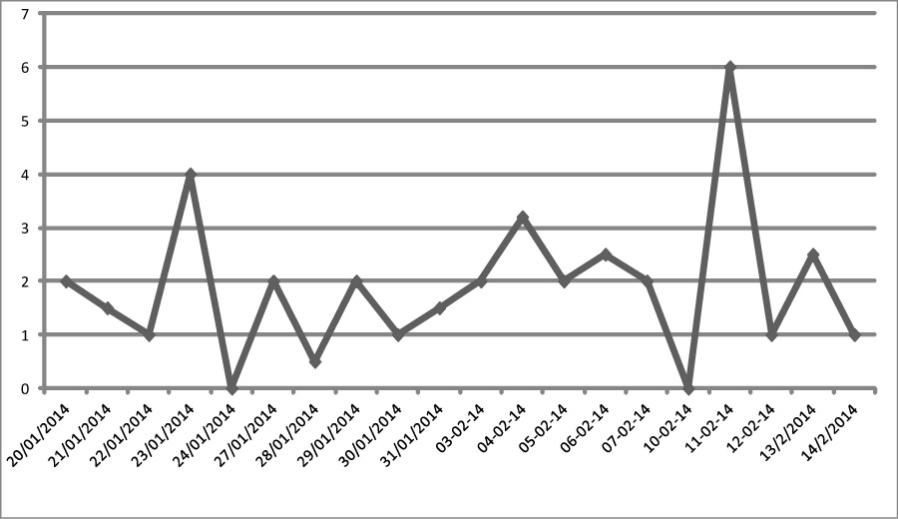
The average daily power interruption rate in the four hospitals of Addis Ababa (January-February, 2014). Fluctuations were measured during work hours (8 hours) and days.

During the study period, the power fluctuation frequency in the study hospitals was monitored for one month. In one of the hospitals, the power supply was too weak to run the computers and the EMR was not functional during the study period. In the other hospitals, the daily hours of the power interruption were recorded, and the average of the 4 hospitals is shown in Figure 4. Accordingly, the mean time the power was interrupted for was 1.7 hours per day (SD 0.3). Of all the hospitals, 3 had a standby generator, but the generators could only reach the emergency and surgical departments and hence could not support the full running of the EMR in all of the departments.

#### Determinants of EMR User Satisfaction

In the binary logistic regression analysis, the following were found to be significantly associated with EMR satisfaction (Table 7): computer access method in the hospital, IT qualification, EMR use, training, perceived system quality, perceived information quality, and perceived service quality. Respondents who reported to have good IT qualification were 3 times (adjusted OR 3.21, 95% CI 3.05-8.12) more likely to be satisfied with EMR systems when compared with those reported not having IT qualification, and those who had individual computer access were 4 times (adjusted OR 4.10, 95% CI 2.85-21.95) more likely to use the EMR than those who shared the computer with more than 5 people. Respondents who were currently using the system were 8 times more likely to be dissatisfied (adjusted OR 7.99, 95% CI 5.62-9.10), and those who received initial EMR training were 3 times (adjusted OR 3.04, 95% CI 2.05-8.12) more likely to be satisfied with EMR systems. The respondents who perceived the system to be of good quality were 2 times (adjusted OR 2.2 95% CI 1.34-3.09) more likely to be satisfied with the EMR, while those who perceived the information to be of good quality (adjusted OR 1.94, 95% CI 1.12-3.23) and those who perceived service to be of good quality (adjusted OR 8.23, 95% CI 3.23-17.01) were 2 and 8 times more likely to be satisfied, respectively. The result of respondent characteristics and its associated factors are shown in Table 7.

**Table 7:** Binary logistic regression analysis of factors associated with EMR satisfaction with a 95% CI and a significance level of *P*<.05 (n=309).

Characteristics		EMR satisfaction, n (%)		OR (95% CI)	AOR^a^ (95% CI)
		Dissatisfied	Satisfied		
**Computer access in hospital**					
	Individual	18 (21.6)	65 (78.3)	17.77 (12.62-26.42)	4.10 (2.85-21.95)
	2 practitioners	27 (57.4)	20 (42.5)	11.85 (2.53-55.34)	2.91 (1.94-6.13)
	3 practitioners	51 (75.0)	17 (25.0)	5.33 (1.15-24.64)	1.5 (0.10-2.25)
	4 practitioners	66 (91.6)	6 (8.4)	1.45 (0.27-7.61)	1.11 (0.18-2.37)
	>5 practitioners	32 (94.1)	2 (5.9)	1.0	1.0
**IT qualification**					
	None	70 (86.4)	11 (13.6)	1.0	1.0
	Reasonable	77 (60.1)	51 (39.9)	4.21 (2.03-8.72)	2.11 (1.58-8.77)
	Good	52 (52.0)	48 (48.0)	5.87 (2.78-12.39)	3.21 (3.05-8.12)
**SmartCare training**					
	Yes	109 (53.1)	96 (46.9)	8.01(18.56-27.78)	3.04(2.31-7.34)
	No	89 (86.4)	14 (13.6)	1.0	1.0
**Current SmartCare use**					
	Yes	12 (12.2)	86 (87.8)	11.8 (6.68-26.86)	7.89 (3.62-9.10)
	No	187 (88.6)	24 (11.4)	1.0	1.0
**Perceived system quality**					
	Good	160 (68.9)	72 (31.1)	3.21 (1.34-4.23)	2.2 (1.34-3.09)
	Not good	23 (29.8)	54 (69.2)	1.0	1.0
**Perceived information quality**					
	Good	140 (65.1)	75 (34.9)	2.8 (1.23-3.78)	1.94 (1.12-3.23)
	Not good	32 (34.0)	62 (66.0)	1.0	1.0
**Perceived system quality**					
	Good	73 (90.1)	8 (9.8)	9.34 (4.23-18.34)	8.23 (3.23-17.01)
	Not good	197(86.4)	31(13.6)	1.0	1.0

^a^Adjusted OR

### Unexpected Observations

There was one unexpected observation we want to point out. There was no dedicated information communication technology (ICT) support center in all of the hospitals despite the implementation of such an expensive server and network infrastructure. Of all 5 hospitals, 3 did not even have any professional IT technical support. The other 2 hospitals did have professional IT support; however, they were not primarily responsible for the EMR. The country is indeed training health information technicians at both the diploma and masters level to manage such systems but there were no health information technicians hired in all of the hospitals. Additionally, we observed that the technical support from TUTAPE was not sufficient during the study duration. The technical support team usually took 2-3 days to visit the hospital and solve the problem.

## Discussion

### Principal Findings

The purpose of the study was to assess the use and the determinant factors of user satisfaction in an implemented EMR system through a comprehensive assessment of usage patterns, user satisfaction, and determinant factors which affect the EMR system. This study had four main results.

First, the usage of the system was found to be low. To increase the use of the system, most of the physicians expressed the need to hire secretaries as the nurses expressed a lack of time to input information and the proper maintenance of computers, and the laboratory and pharmacy staff complained about the lack of use of the system by other departments. This result is not actually surprising given that health professionals are expected to do dual documentation both on the computer and on paper which makes them feel that transferring data to the EMR is not their duty. Hence, most of the doctors and the nurses were complaining about the lack of time and most of them demanded secretaries to be hired so that the secretary can transfer the paper documentation to the EMR. The other aspect of the challenge is the partial use of the system in the hospital departments. Hospital work flows are interconnected, in which the activity of one department affects the other. Therefore, there is a need to implement the system to interdependent departments especially to those that are pillars of the hospital system (eg, laboratory, pharmacy, and radiology).

Second, the result of this study shows that only 2 of the core EMR functions were frequently used, 3 of the functionalities were never used, and participants were also unaware of 4 out of 12 core EMR functions. The participants enrolled in this study were all employed for >6 months. Thus, they were likely to be familiar with the main system functionalities. The low rate of use and awareness might be attributed to the general low adaptation rate of the EMR in the hospital and the training quality.

Third, the user satisfaction of the respondents was also found to be low. The majority of them reported to be dissatisfied with the use of the system. The main reported reason of the dissatisfaction was the service quality in the hospital. This was mainly due to lack of IT support, the shared computer access, and frequent power interruption.

Fourth, the user satisfaction was strongly correlated with service quality and system use. It was also moderately correlated with IT qualification, computer access method, perceived system, and information quality. Given the infrastructural and organizational challenges, such a strong correlation between service quality and use and user satisfaction was expected. However, the level of strength of the relationship was high, which shows that there was a need to improve the service quality and the current way of using the EMR in the hospitals.

### Study Strengths and Weaknesses

Totally, 406 professionals (96.2% response rate) from 5 hospitals participated in this study. The response rate was very high when compared to other evaluation studies, which can be attributed to the use of data collectors from each hospital and our encouragement for participation by providing rewards. We addressed different potential system users by including physicians, nurses, laboratory, pharmacy, and HMIS staff.

There are some limitations to this study. Firstly, our data collection period was short (1 month). As pointed out by Meijden [3], system implementation is dynamic and the level of use rate fluctuates over time. Hence, this result may not exactly reflect the current status of the EMR implementation in those hospitals. The second limitation is with respect to the hypothesized determinant factors. This study is only based on the six constructs of the D&M model but there are many other organizational and human factors which affect acceptance and thus implementation success of an EMR system. Future studies can include those additional variables to have a complete picture of EMR success in those settings.

### Results in Relation to Other Studies

There are different evaluation studies regarding the use and user satisfaction of health professionals with respect to EMR systems and different adoption rates were reported. For example, Laerum et al [34] reviewed EMR system use in 19 hospitals and reported that system use was low and only 2 out of 7 implemented functions were frequently used, which is in line with our results. Another similar study in Saudi Arabia [9] also reported that the use of different core EMR components were minimal. In that study, 54.9% of the physicians never used ≥1 of the 10 investigated core EMR functions. Mikkelsen et al also assessed the challenges of parallel documentation (EMR and paper-based records) and found that it is a source of dissatisfaction and inconsistency, which is similar to our study [35].

Alharthi et al [36] assessed physicians’ satisfaction of an EMR system and reported only 40% of them were satisfied. A similar low satisfaction rate was reported in Malaysia [37], Oman [38], and Kenya [39] which all is similar to our result. However, a recent study by Jia-lin [40] in two big hospitals in China reported a satisfaction rate of 70.7%. This difference might be because of the infrastructural differences in the study setting hospitals. Another study by Palm et al [5] reported that medical secretaries were more satisfied than nurses and physicians and Moody et al [41] and others [35,39,40] reported that nurses were more satisfied than physicians with the use of EMR, which is similar to our result.

Common in most studies and in our study are the factors that affect the success of implementation of an EMR system. Palm et al [5], in his assessment of determinants of user satisfaction of EMR systems, reported that female gender, perceived system quality, usefulness, and service quality are strongly correlated with satisfaction. Similarly, another study by Chatzoglou et al [27] reported that user background, information quality, and service quality directly and positively affect user satisfaction. Consistent with many evaluation studies and models [3,5,12,26,42-44], system quality, information quality, and service quality are determinant factors for user satisfaction. However, in our study we found out that there was a strong correlation between user satisfaction and system quality. This difference might be due to the infrastructural challenge in our study hospitals in which there were frequent power interruptions and no dedicated ICT support centers.

We agree with Meijden [3] that the D&M’s conceptual framework does not address different contingent factors for the success of an EMR system. In our study, user IT qualification and computer access methods were found to be significant determining factors but we were not able to accommodate them in the framework. These are also reported as determinant factors in other studies [9,45], but computer access method was a significant determinant factor in our study. We believe that this factor is significant for low-resource settings, given that most clinicians (43% of the physicians and 31% of nurses) shared one computer for ≥4 people. .

### Meaning and Generalizability

Even though many hospitals implemented an EMR system in developing countries, very few evaluation studies exist on use, user satisfaction, and factors affecting it. In this study we attempted to close this gap by assessing use and user satisfaction in low-resource setting hospitals, and we believe that the result will be helpful to health care managers and decision makers as an input for future EMR implementation or expansion projects. The ministry of health of Ethiopia plans to expand the EMR to all other hospitals, and we are hopeful that this study will help them as an input. As outlined above, our result shows that more emphasis must be given to service quality in implementing the EMR system to the other hospitals. Since our study includes both teaching and general hospitals as well different professionals in the hospitals, we believe that our findings are generalizable to other similar setting hospitals in developing countries.

### Unanswered and New Questions

The informatics community perceives the D&M model as the best and most validated model to measure the success of an implemented information system [32,43,46-49]. However, as also stated by Meijden [3], the D&M’s conceptual framework does not address all information system success factors. In our study results, we were unable to categorize the computer skill and experiences into the model. Hence, a more comprehensive model, which takes into account the different factors in low-resources setting hospitals, is necessary.

The other necessary but unaddressed aspect of the evaluation is the financial feasibility of the system. In the study hospitals, the system was implemented by external NGO-funding that provided a higher budget for EMR implementation than governmental funding. Therefore, we recommend the Ministry of Health to conduct more comprehensive, cost-benefit analyses which include a qualitative evaluation on the system before implementing the system to the other hospitals.

### Conclusions

Health professionals’ use of the EMR is low and they are generally dissatisfied with the service of the implemented system. The result of this study showed that the dissatisfaction was caused mainly and strongly by the poor service quality, the current practice of double documentation (EMR and paper-based), and partial departmental use of the system in the hospitals. Thus, future interventions to improve the current use or future deployment projects should focus on improving the service quality, such as power infrastructure, user support, trainings, and more computers in the wards. After service quality improvement, other departments (especially inter-dependent departments) may be motivated and supported to use the EMR to avoid the dependency deadlock. Further evaluation studies that include a cost-benefit analysis are recommended.

## Abbreviations

D&M: DeLone and MacLean
EMR: Electronic medical record
HMIS: Health management information system
ICT: Information communication technology
IQR: Interquartile range
IRB: Institute Review Board
IT: Information technology
NGO: Non governmental organization
